# Clinical and Radiologic Outcomes of Bioinductive Collagen Implant Augmentation in Sugaya Type III Rotator Cuff Retears

**DOI:** 10.3390/diagnostics16111710

**Published:** 2026-06-02

**Authors:** Daehee Lee, Jaewook Park, Jaesung Yoo

**Affiliations:** 1 Department of Orthopaedic Surgery, WELIVE Hoispital, Asan 31465, Republic of Korea; osdankook@dkuh.co.kr (D.L.); jaewookpark@dkuh.co.kr (J.P.); 2 Department of Orthopaedic Surgery, Dankook University Hospital, Cheonan 31116, Republic of Korea

**Keywords:** rotator cuff injuries, reoperation, arthroscopy, collagen, bioprosthesis

## Abstract

**Background**: Sugaya type III rotator cuff re-tears are characterized by preserved tendon continuity with reduced thickness and are often associated with persistent pain and functional impairment. Bioinductive collagen implants may enhance tendon healing, but clinical evidence in this population remains limited. This study aimed to evaluate the clinical and radiologic outcomes of arthroscopic repair with bioinductive collagen implant augmentation in patients with Sugaya type III re-tears. **Methods**: This retrospective case series (Level IV) included 15 patients (mean age 61.7 years) with MRI-confirmed Sugaya type III re-tears. An a priori power analysis based on a large effect size (Cohen’s d = 0.80) indicated that a sample size of 15 would provide 80% power to detect clinically meaningful changes in the primary endpoint. Clinical outcomes were assessed preoperatively and at 6 and 12 months postoperatively using VAS, ASES, SANE, and WORC scores. MRI was used to evaluate changes in supraspinatus tendon thickness. Non-parametric statistical analysis with Bonferroni correction was applied. **Results**: The median VAS pain score improved from 6.5 (IQR, 6.0–7.0) preoperatively to 2.8 (IQR, 2.0–3.5) at 6 months and to 2.1 (IQR, 1.5–2.8) at 12 months (adjusted *p* < 0.001). The median ASES score increased from 45.0 (IQR, 39.0–51.0) to 78.0 (IQR, 72.0–85.0), with a median improvement of 33 points. SANE and WORC scores also showed significant improvements. Supraspinatus tendon thickness increased from 4.8 mm (IQR, 3.7–5.7) to 6.9 mm (IQR, 5.4–8.3) at 12 months (adjusted *p* < 0.001). No graft failure was observed on follow-up MRI. **Conclusions**: Arthroscopic repair with bioinductive collagen implant augmentation may be associated with short-term improvements in pain, function, and tendon thickness in patients with Sugaya type III re-tears. Given the small sample size and lack of a control group, these findings should be interpreted cautiously, and further prospective comparative studies are needed.

## 1. Introduction

Rotator cuff tears are one of the primary causes of shoulder pain and functional impairment, and their incidence is gradually increasing due to an aging population [1]. In addition to mechanical factors, systemic and metabolic conditions such as diabetes and smoking have been reported to adversely affect tendon healing and repair integrity [2]. Several recent studies have shown an association between rotator cuff tear incidence and thyroid disorders, highlighting the influence of metabolic factors on tendon biology [3]. Arthroscopic rotator cuff repair is widely performed as a standard treatment; however, postoperative re-tears are still reported at relatively high rates and negatively impact patient prognosis [4].

In the Sugaya classification, type III is defined as a condition in which the supraspinatus tendon maintains continuity but demonstrates reduced thickness compared to normal [5]. Although tendon continuity is preserved, this pattern may reflect compromised structural integrity and altered biomechanical properties, potentially contributing to persistent symptoms. Indeed, some patients experience prolonged pain and functional impairment despite preserved continuity on imaging [6].

When considering reoperation in patients with incomplete healing or tendon thinning, such as Sugaya type III lesions, previous studies have reported higher re-rupture rates after revision surgery compared with primary repair [7]. In addition, prolonged rehabilitation after revision repair may place a considerable burden on both patients and surgeons. Therefore, identifying alternative treatment strategies for symptomatic Sugaya type III lesions remains clinically relevant. In this setting, established clinical guidelines for rotator cuff management emphasize the importance of patient-specific factors, including tendon quality and comorbid conditions, in determining optimal treatment strategies [8].

The bioinductive collagen implant (REGENETEN^®^) is an absorbable collagen scaffold designed to enhance the biological healing environment by promoting fibroblast proliferation and facilitating neo-tendon-like tissue formation, rather than providing direct mechanical reinforcement [9,10]. Clinical studies in partial- and full-thickness rotator cuff tears have reported improvements in pain, function, and tendon thickness with low complication rates [11,12]. However, evidence regarding its application specifically in patients with Sugaya type III re-tears is limited.

In this study, we hypothesized that arthroscopic re-suturing combined with bioinductive collagen implant augmentation would be associated with improvements in clinical outcomes and supraspinatus tendon thickness in patients with symptomatic Sugaya type III re-tears. The primary endpoint was the improvement in patient-reported clinical outcomes at 12 months, and secondary endpoints included changes in MRI-based supraspinatus tendon thickness and range of motion. Therefore, the objective of this study was to evaluate the short-term clinical and radiologic outcomes following the application of bioinductive collagen implant augmentation in patients with Sugaya type III rotator cuff re-tears.

## 2. Methods

This study was a retrospective case series (Level IV) conducted at a single institution. The study protocol was reviewed and approved by the Institutional Review Board (IRB No. DKUH 2026-01-029) prior to initiation. Written informed consent for participation and publication of anonymized clinical data was obtained from all patients. All procedures were performed in accordance with the ethical standards of the institutional and national research committee and with the 1964 Helsinki Declaration and its later amendments. The study included 15 patients who underwent rotator cuff reoperation at our hospital between December 2023 and October 2024 and were diagnosed with Sugaya type III re-tear on MRI. Sugaya type III re-tears were diagnosed based on standard MRI criteria according to the original Sugaya classification system, defined as maintained tendon continuity with thinning of the repaired tendon compared with the expected normal thickness. Classification was determined by consensus between an experienced orthopedic surgeon and a musculoskeletal radiologist based on postoperative MRI findings. No absolute thickness cutoff value was used for classification purposes [5].

Inclusion criteria were (1) adults aged 18 years or older, (2) Sugaya type III re-tear with persistent pain for over 2 years post-surgery, and (3) ability to undergo at least 12 months of follow-up. Exclusion criteria were (1) Sugaya type IV or V, (2) shoulder joint arthrosis classified as Hamada grade III or higher, and (3) concomitant infection.

To evaluate whether the sample size was statistically adequate for the primary hypothesis, a power analysis was performed for the within-subject comparison of preoperative and 12-month postoperative ASES scores using a two-sided paired test with an alpha level of 0.05. Based on a reported minimal clinically important difference (MCID) of 27.1 points for the ASES score following arthroscopic rotator cuff repair [13], and assuming a large effect size (Cohen’s d = 0.80), a total of 15 patients would provide 80% power.

This analysis was performed for the primary endpoint only and did not account for multiple secondary outcomes. Given the retrospective design of the study, the sample size was not prospectively determined; rather, the final study group included all eligible patients during the study period, and the power analysis was conducted post hoc to assess statistical adequacy.

This study was designed as a retrospective single-arm case series without a control group. Therefore, the independent effect of the bioinductive collagen implant cannot be separated from other surgical procedures performed at the same time or from the natural postoperative course. The primary purpose was to evaluate short-term clinical and radiologic outcomes and the feasibility of this approach rather than to establish relative effectiveness.

### 2.1. Surgical Technique

All procedures were performed by a single surgeon under interscalene block anesthesia without general anesthesia. The patient underwent surgery in the beach-chair position.

First, diagnostic arthroscopy was performed via a posterior approach to assess the rotator cuff condition and associated pathology, followed by the creation of standard anterior and lateral access points. If arthroscopic examination revealed that the coracoacromial (CA) ligament was not released, or that the inferior border of the acromion was irregular or rough, CA ligament release and acromioplasty were additionally performed to prevent impingement.

**Figure 1:**
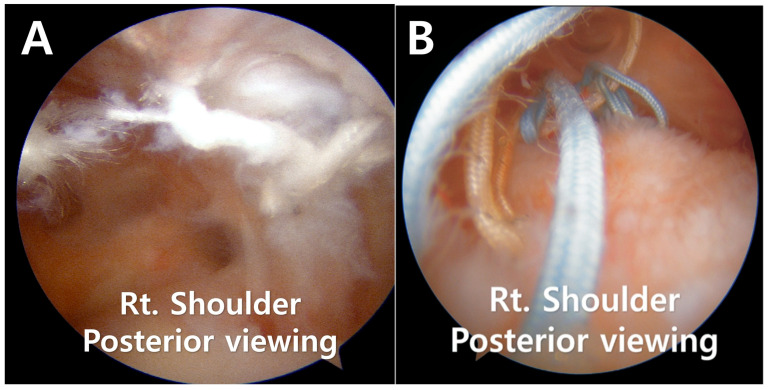
Arthroscopic findings of the right shoulder demonstrating loosening of previously used FiberWire sutures. (**A**) In-tra-articular view showing multiple loosened FiberWire sutures and suture knots from the previous rotator cuff repair. The displaced sutures failed to maintain adequate mechanical stability of the repaired tendon. (**B**) Subacro-mial view demonstrating loosened and displaced FiberWire sutures and knots around the rotator cuff footprint, which may contribute to mechanical irritation within the subacromial space. All loosened FiberWire strands and unnecessary knots were carefully removed during revision surgery.

The suture knots and fiber wires used in the previous surgery were loosened in multiple areas, which not only failed to provide mechanical stability but could also cause collision and irritation. Therefore, all loosened fiber wires and unnecessary suture knots were removed whenever possible (Figure 1).

Subsequently, the bioinductive collagen implant (REGENETEN^®^, Smith & Nephew, Andover, MA, USA; bioinductive collagen implant, FDA-cleared for rotator cuff augmentation) was inserted through the lateral insertion port using the manufacturer’s dedicated delivery system. Prior to insertion, the bleeding tissue on the cuff surface (bursal surface bleeding bed) was cleared using a shaver and RF probe to ensure close adherence of the scaffold to the tendon.

**Figure 2:**
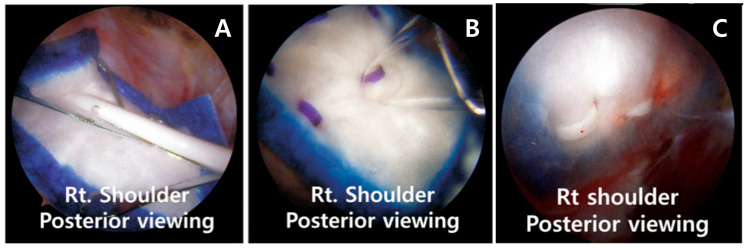
Arthroscopic views demonstrating the placement and fixation of the REGENETEN bioinductive collagen implant: (**A**) The implant was positioned to completely cover the thinned and heterogeneous supraspinatus tendon, extending medially to the musculotendinous junction and laterally to the greater tuberosity footprint, with at least a 5 mm overlap beyond the anterior and posterior tendon margins. (**B**) Arthroscopic image after fixation of the REGENETEN implant using tendon anchors, showing stable adaptation of the graft to the underlying cuff surface. (**C**) Final arthroscopic appearance after completion of bone anchor fixation at the greater tuberosity, demonstrating stable fixation and complete adaptation of the implant to the underlying cuff surface.

The implant was positioned to fully cover the thinned and heterogeneous region of the supraspinatus tendon and was extended medially to the musculotendinous junction and laterally to the greater tuberosity footprint. The implant coverage was also extended anteriorly and posteriorly to secure a margin of at least 5 mm beyond the border of the thinned tendon, and the graft was positioned to adequately overlap the healthy tendon (Figure 2A).

Fixation was achieved using a combination of bioabsorbable staples and tendon anchors. Staples were inserted at regular intervals (approximately 5–7 mm) along the medial and anteroposterior edges of the implant to ensure even adherence of the scaffold to the cuff surface (Figure 2B,C). During this step, precautions were taken to prevent staple protrusion or implant wrinkling.

At the final arthroscopic examination, the implant was observed to be securely attached to the cuff surface. During joint movement, neither displacement nor lifting of the graft was observed. After confirming that the implant was evenly positioned, securely fixed, and stable on the cuff surface, the surgery was completed.

### 2.2. Postoperative Rehabilitation

Following surgery, the patient wore a shoulder abduction sling for 48 h and thereafter was instructed to wear it as required for up to two weeks. During the initial two weeks, shoulder joint movement was restricted, permitting only light movement of the wrist, fingers, and elbow joints. From two weeks onwards, passive range of motion (ROM) exercises commenced under the guidance of a physiotherapist, with external rotation and forward elevation beyond 90° restricted. From four weeks onwards, the range of external rotation was progressively increased within pain-free limits.

From the 6-week mark, active-assisted range of motion (ROM) exercises and scapular stabilization exercises commenced, alongside isometric muscle contraction exercises to maintain baseline strength in the rotator cuff and surrounding muscles. Subsequently, depending on the patient’s recovery progress, this transitioned to active ROM exercises, with the goal for most patients being full restoration of joint range of motion within 12 weeks.

After three months, progressive muscle strengthening exercises using elastic bands and light dumbbells commenced. This phase focused on strengthening the rotator cuff muscle group and scapular stabilizing muscles. Depending on the patient’s condition, light domestic activities and a return to work were permitted.

After six months, patients were gradually permitted to resume daily activities and sports. High-intensity resistance training and overhead sports activities were progressively allowed between nine and twelve months. Ultimately, all patients resumed activities based on pain-free joint movement, restored muscle strength, and functional stability.

### 2.3. Evaluation of Clinical and Radiologic Outcomes 

Clinical evaluation was conducted at preoperative, 6-month, and 12-month postoperative time points. Pain was assessed using the visual analogue scale (VAS) from 0 (no pain) to 10 (extreme pain). Functional evaluation utilized the American Shoulder and Elbow Surgeons (ASES) score, the Single Assessment Numeric Evaluation (SANE) score, and the Western Ontario Rotator Cuff (WORC) index. Furthermore, the range of motion (ROM) was measured for forward flexion, external rotation at the side, and internal rotation at the spinal level. Range of motion (ROM) was assessed by the treating surgeon, and all measurements were recorded at the maximum pain-free range. ROM measurements were performed by the treating surgeon, which may introduce measurement bias. No independent blinded assessor was involved in the clinical evaluation. Patient-reported outcome measures are inherently subject to response and expectancy bias, particularly in the revision surgery setting. These potential sources of bias were not controlled in the present retrospective design. In addition, test–retest reliability and measurement stability parameters (e.g., minimal detectable change or standard error of measurement) were not assessed within this dataset, which may limit the precision and interpretability of the clinical outcome measurements.

Radiologic evaluation was performed using MRI at preoperative and 12-month postoperative time points. All MRI scans were acquired using the same equipment (3.0-T MRI scanner; Philips Ingenia, Philips Healthcare, Best, The Netherlands) and a standardized protocol, including T2-weighted fat-suppressed coronal oblique sequences. Supraspinatus tendon thickness was measured on T2-weighted coronal oblique images using a standardized method, consistent with previously reported techniques [14]. Sugaya type III was defined as a reduction in thickness to 25% or less relative to the contralateral side, or an absolute thickness of 6.0 mm or less when no image was available on the affected side. MRI measurements were independently repeated twice by an orthopedic surgeon (with 15 years’ experience) and a radiologist (with 9 years’ experience), with the mean value used for analysis. Intra-observer and inter-observer reliability were assessed by calculating the intraclass correlation coefficient (ICC). Graft failure was defined as graft loss, re-rupture, or patch detachment on MRI. The presence of complications (infection, stiffness, and nerve injury) was also investigated in all patients.

Complications were prospectively recorded during scheduled follow-up visits. Postoperative stiffness was defined as forward flexion < 120° or external rotation < 30° at 6 months postoperatively. Other adverse events, including infection, nerve injury, implant-related complications, and re-tear, were systematically assessed through clinical examination and follow-up MRI. All adverse events were documented regardless of severity.

### 2.4. Statistical Analysis

Continuous variables are reported as median (interquartile range [IQR]), and categorical variables as counts and percentages. Data normality was assessed using the Shapiro–Wilk test. Given the small sample size (*n* = 15), comparisons between preoperative and 12-month postoperative values were performed using the Wilcoxon signed-rank test. Inter-observer and intra-observer reliability for MRI measurements were assessed using the intraclass correlation coefficient (ICC) based on a two-way random-effects model with absolute agreement. Because multiple outcomes were tested, Bonferroni correction was applied to control for multiplicity, and adjusted *p*-values are reported where appropriate. A two-sided *p* value < 0.05 was considered statistically significant. All analyses were performed using IBM SPSS Statistics for Windows, Version 21.0 (IBM Corp., Armonk, NY, USA). The primary endpoint was the change in ASES score at 12 months.

## 3. Results

### 3.1. Demographics

A total of 15 patients were included in this study, and the average age was 61.7 years (range, 55–70 years). The participants included 8 males (53.3%) and 7 females (46.7%), and the average BMI was 24.6 ± 1.7 kg/m^2^. Dominant-side lesions appeared in 9 cases (60.0%). The average symptom duration was 2.7 ± 0.4 years, and all patients had Sugaya type III re-tears with persistent pain for over 2 years after surgery. Diabetes was observed in 4 cases (26.7%), and a smoking history was found in 6 cases (40.0%) (Table 1).

### 3.2. Clinical Outcomes

**Figure 3:**
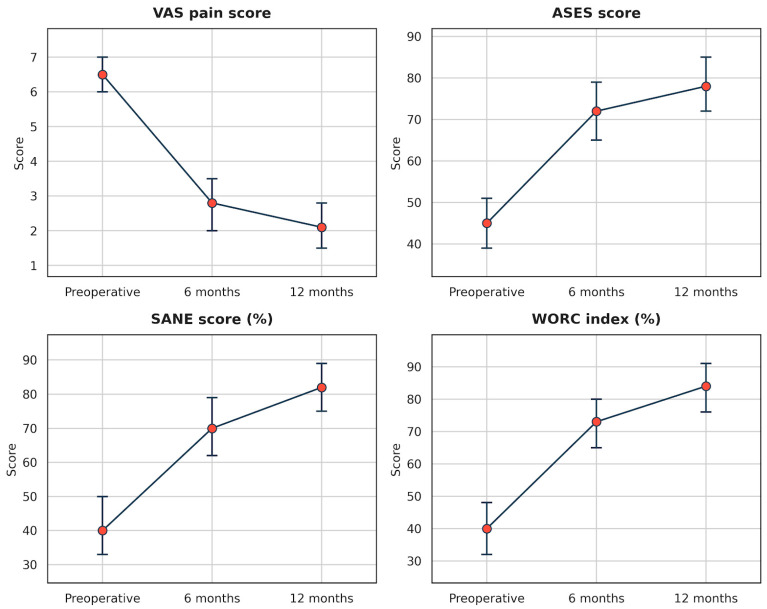
Changes in clinical outcomes across the preoperative, 6-month, and 12-month follow-up time points. The line graphs illustrate the group-level trajectories for the Visual Analog Scale pain score, American Shoulder and Elbow Surgeons score, Single Assessment Numeric Evaluation score, and Western Ontario Rotator Cuff (WORC) index. Data points represent the median values.

Significant improvements were observed in patient-reported outcome measures at both 6 and 12 months postoperatively. The median VAS pain score decreased from 6.5 (IQR, 6.0–7.0) preoperatively to 2.8 (IQR, 2.0–3.5) at 6 months and 2.1 (IQR, 1.5–2.8) at 12 months (adjusted *p* < 0.001). The median ASES score improved from 45.0 (IQR, 39.0–51.0) preoperatively to 78.0 (IQR, 72.0–85.0) at 12 months, representing a median increase of 33 points. Similarly, the SANE score increased from 40.0% (IQR, 33.0–50.0) to 70.0% (IQR, 62.0–79.0) at 6 months and 82.0% (IQR, 75.0–89.0) at 12 months (adjusted *p* < 0.001). The WORC index showed a significant improvement from 40.0% (IQR, 32.0–48.0) preoperatively to 73.0% (IQR, 65.0–80.0) at 6 months and 84.0% (IQR, 76.0–91.0) at 12 months (adjusted *p* < 0.001) (Table 2, Figure 3).

**Figure 4:**
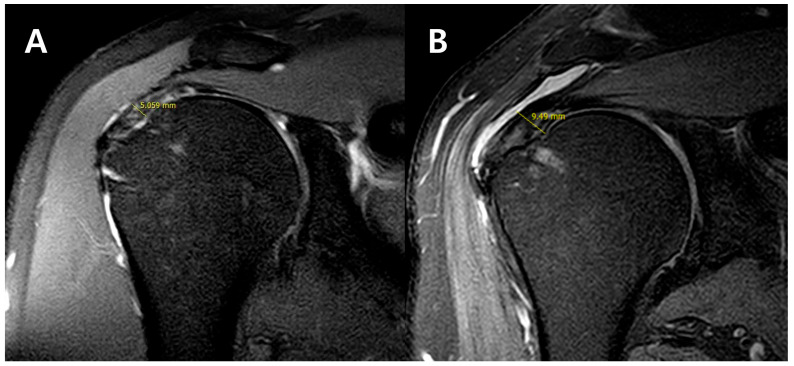
T2-weighted magnetic resonance imaging (MRI) of a 57-year-old male patient who underwent rotator cuff repair at another institution 3 years earlier: (**A**) Preoperative MRI demonstrating more than a 25% reduction in supraspinatus tendon thickness compared with the contralateral side, with a measured thickness of 5.059 mm. (**B**) Postoperative MRI obtained 1 year after surgery showing an increased supraspinatus tendon thickness of 9.49 mm.

MRI evaluation revealed that supraspinatus tendon thickness increased from 4.8 mm (IQR, 3.7–5.7) preoperatively to 6.9 mm (IQR, 5.4–8.3) at 12 months (adjusted *p* < 0.001). The implant remained well-positioned in all patients, and no graft failure was observed on the 12-month follow-up MRI (0%) (Table 2, Figure 4). For MRI-based supraspinatus thickness measurements, inter-observer reliability showed an ICC of 0.93 (95% CI, 0.84–0.97), and intra-observer reliability showed an ICC of 0.94 (95% CI, 0.87–0.98), indicating excellent measurement reproducibility.

### 3.3. Range of Motion

Anterior elevation range of motion improved from 162° (IQR, 155–170) preoperatively to 176° (IQR, 171–180) at 12 months (adjusted *p* = 0.037). External rotation increased from 60° (IQR, 55–65) preoperatively to 71° (IQR, 66–76) at final follow-up (adjusted *p* = 0.041). Internal rotation improved from a median spinal level of L3 (IQR, L2–T12) preoperatively to T11 (IQR, T12–T10) at 12 months (adjusted *p* = 0.018) (Table 2).

### 3.4. Complications

No postoperative complications were observed during the follow-up period. There were no cases of infection, nerve injury, implant-related complications, or structural failure. Additionally, no patients met the predefined criteria for postoperative stiffness. All patients completed follow-up without any reported adverse events.

Due to the small sample size, graphical representation with error bars was not included, and outcomes are presented using median and interquartile ranges to reflect the data distribution.

## 4. Discussion

The hypothesis of this study was that arthroscopic re-suturing combined with bioinductive collagen implant augmentation would improve clinical and radiologic outcomes in patients with Sugaya type III rotator cuff re-tears. In this retrospective case series, short-term improvements in clinical scores and tendon thickness were observed; however, these findings should be interpreted as feasibility and early outcome data rather than proof of treatment superiority. Significant improvements were observed in patient-reported outcome measures, including VAS, ASES, SANE, and WORC scores at final follow-up. In addition, supraspinatus tendon thickness increased from a median of 4.8 mm (IQR, 3.7–5.7) preoperatively to 6.9 mm (IQR, 5.4–8.3) at 12 months. The observed increase in supraspinatus tendon thickness may be clinically relevant, as previous studies have reported that increased tendon thickness on MRI is associated with improved structural integrity and tendon healing following rotator cuff repair [14,15]. However, a universally accepted minimal clinically important difference for MRI-measured tendon thickness has not been established, and therefore, the clinical significance of this change should be considered preliminary.

Sugaya type III is radiologically defined as preservation of tendon continuity with reduced thickness [14]. Although tendon continuity is preserved, this pattern may reflect compromised structural integrity and altered biomechanical properties. Prior studies have reported that postoperative tendon elongation and tension loss may contribute to persistent pain and functional limitations despite structural continuity on MRI [15,16]. Clinically, patients with Sugaya type III lesions often experience prolonged discomfort and suboptimal functional recovery [17].

In contrast, Sugaya type IV–V lesions represent clear structural re-tears, for which revision surgery is commonly considered when symptoms persist [14,18,19]. Management of type III lesions is more complex, and tissue quality deterioration and high re-tear rates following revision repair have been reported [17,19,20], making surgical indications controversial.

Current clinical guidelines for rotator cuff management emphasize the importance of tendon quality, patient-specific factors, and comorbid conditions when determining optimal treatment strategies, particularly in revision settings [8]. Therefore, the use of biological augmentation may be considered in selected patients with compromised tendon integrity.

Several recent studies investigating bioinductive collagen implants in rotator cuff tears have shown favorable clinical outcomes, increased tendon thickness, and low failure rates [21,22,23]. Sugaya type III shares pathophysiological characteristics with high-grade partial-thickness tears, as both conditions involve preservation of continuity with compromised thickness and tension [24]. Based on this rationale, bioinductive collagen implant augmentation was applied in these patients.

In revision rotator cuff repair settings, the biologic healing environment is often compromised due to prior surgical intervention, fibrotic remodeling, and reduced vascularity of the tendon tissue. Bioinductive collagen scaffolds are designed to help promote tendon regeneration through fibroblast proliferation, collagen deposition, and neotendon formation. In addition to providing a structural matrix, these scaffolds may influence load-sharing and stress distribution across the repair site, potentially mitigating excessive strain on the residual tendon. These concepts are supported by recent literature on tendon structure and load remodeling, which emphasizes the dynamic interaction between biological tissue formation and mechanical loading conditions [25]. Several recent studies have suggested that scaffold-mediated tissue formation may contribute to improved tendon thickness and structural continuity, although the precise biological mechanisms are still not fully understood [9,10].

The observed improvements in clinical scores and radiologic thickness, together with the absence of graft failure at 12 months, suggest that this combined surgical approach may help improve short-term structural and symptomatic outcomes in selected patients. However, given the absence of a comparator group, the independent contribution of the implant cannot be separated from other procedures performed at the same time, such as debridement or acromioplasty. This represents a major limitation of our study, as the independent effect of the bioinductive implant cannot be isolated. Rather than focusing solely on structural continuity, the goal in treating Sugaya type III may involve optimizing tendon quality and functional capacity; however, causal inferences regarding mechanisms cannot be drawn from this study design.

This study has several limitations. First, the small sample size (n = 15) limits the statistical power and generalizability of the findings, and the assumption of a large effect size in the power analysis may be considered optimistic. Second, the retrospective design and absence of a control group limit the ability to establish comparative effectiveness and introduce the possibility of selection bias, as only patients deemed suitable for this approach were included. Importantly, the independent effect of the bioinductive collagen implant cannot be isolated from other surgical procedures performed at the same time, including debridement, removal of loose hardware, and possible acromioplasty. This represents a major limitation of our study and restricts causal interpretation of the observed improvements. Third, the follow-up period of 12 months is relatively short for evaluating long-term structural durability and clinical outcomes. Fourth, although excellent inter- and intra-observer reliability was observed for MRI measurements, inherent limitations of radiologic assessment and potential observer bias cannot be completely excluded. In addition, no blinded assessor was used for clinical evaluation, and test–retest reliability of the clinical outcome measures was not assessed, which may introduce measurement bias. Furthermore, test–retest reliability and measurement stability parameters, such as MDC or SEM, were not evaluated, which may affect the interpretability of observed changes in clinical scores. Factors such as diabetes and smoking, which were common among our patients, are known to negatively affect tendon healing through mechanisms such as impaired collagen synthesis, altered vascularity, and increased oxidative stress. These factors may influence the biological integration of collagen scaffolds and the overall healing response. Previous studies have shown that metabolic disorders can lead to structural and compositional changes in tendon tissue, potentially compromising repair outcomes [2,3]. Therefore, the observed clinical improvements in this study should be interpreted within the context of these underlying biological challenges. Finally, patient-reported outcome measures are subject to response bias, and improvements may be influenced by placebo effects or regression to the mean. Furthermore, in the absence of a comparator group or objective return-to-activity metrics, reductions in rehabilitation burden cannot be definitively determined.

Despite these limitations, bioinductive collagen implant augmentation may be a feasible option for selected patients with symptomatic Sugaya type III lesions, particularly when tendon continuity is preserved, but thickness appears compromised. Nevertheless, larger prospective comparative studies with longer follow-up are necessary to confirm the durability, structural integrity, and clinical effectiveness of this approach.

## 5. Conclusions

This retrospective case series evaluated arthroscopic repair combined with bioinductive collagen implant augmentation in patients with symptomatic Sugaya type III rotator cuff re-tears. Significant short-term improvements were observed in pain and functional outcomes compared to preoperative levels. MRI assessment showed increased supraspinatus tendon thickness at 12 months, and no MRI-defined graft failure was identified at final follow-up (0/15).

The present results suggest that this combined surgical approach may be a feasible option in selected patients with symptomatic Sugaya type III lesions. However, given the retrospective design, absence of a control group, and short follow-up duration, these results should be interpreted with caution. Prospective comparative studies with larger sample sizes, longer follow-up, and standardized structural endpoints are necessary to determine the durability and comparative effectiveness of this approach.

## Figures and Tables

**Table 1:** Demographic characteristics.

Case	Age (Years)	Sex	BMI (kg/m^2^)	Dominant Side Involved	Symptom Duration (Years)	Diabetes	Smoking	Supraspinatus Thickness (mm, MRI)
1	62	M	24.8	Yes	2.5	No	No	2.1
2	58	F	22.5	No	2.1	No	No	2.8
3	67	M	26.3	Yes	3.0	Yes	Yes	3.0
4	55	F	23.4	Yes	2.3	No	No	3.7
5	63	M	25.6	No	2.6	No	Yes	4.2
6	60	F	21.8	Yes	2.4	No	No	4.3
7	70	M	27.1	No	3.2	Yes	No	4.8
8	61	M	24.2	Yes	2.8	No	Yes	5.1
9	57	F	23.7	Yes	2.2	No	No	5.2
10	65	M	26.9	No	3.1	Yes	Yes	5.5
11	59	F	22.1	Yes	2.5	No	No	5.7
12	62	M	25.0	Yes	2.7	No	Yes	5.9
13	64	F	23.5	No	2.9	No	No	5.8
14	68	M	27.3	Yes	3.3	Yes	Yes	5.9
15	56	F	22.9	No	2.4	No	No	4.4

All patients met the criteria for Sugaya type III retear based on preservation of tendon continuity with relative thinning compared to the contralateral side on MRI, in accordance with the original Sugaya classification. Values are presented as median (interquartile range) or number (%). BMI = body mass index; M = male; F = female.

**Table 2:** Clinical and radiologic outcomes of the study population (median IQR).

Outcome Measure	Preoperative	Postoperative (6 Months)	Postoperative (12 Months)	Preop vs. 6 Months (adj *p*)	Preop vs. 12 Months (adj *p*)	6 vs. 12 Months (adj *p*)
VAS pain score	6.5 (6.0–7.0)	2.8 (2.0–3.5)	2.1 (1.5–2.8)	<0.001	<0.001	0.032
ASES score	45.0 (39.0–51.0)	72.0 (65.0–79.0)	78.0 (72.0–85.0)	<0.001	<0.001	0.041
SANE score (%)	40.0 (33.0–50.0)	70.0 (62.0–79.0)	82.0 (75.0–89.0)	<0.001	<0.001	0.028
WORC index (%)	40.0 (32.0–48.0)	73.0 (65.0–80.0)	84.0 (76.0–91.0)	<0.001	<0.001	0.036
Supraspinatus thickness (mm, MRI)	4.8 (3.7–5.7)	–	6.9 (5.4–8.3)	-	<0.001	-
Forward flexion ROM (°)	162 (155–170)	173 (168–178)	176 (171–180)	0.021	0.037	0.084
External rotation at side (°)	60 (55–65)	68 (63–73)	71 (66–76)	0.025	0.041	0.092
Internal rotation (spinal level)	L3 (L2–T12)	T12 (L1–T11)	T11 (T12–T10)	0.018	0.018	0.110
Graft failure rate (MRI)	–	–	0% (0/15)	-	-	-

Values are presented as median (interquartile range, IQR). ROM = range of motion; VAS = visual analog scale; ASES = American Shoulder and Elbow Surgeons score; SANE = Single Assessment Numeric Evaluation; WORC = Western Ontario Rotator Cuff Index. No graft failure was detected on MRI at 12 months. Wilcoxon signed-rank test with Bonferroni correction; values represent adjusted *p*-values.

## Data Availability

The datasets generated and/or analyzed during the current study are not publicly available due to patient privacy and ethical restrictions but are available from the corresponding author on reasonable request.
